# A 3-year follow-up case report of rare intramuscular schwannoma of psoas major: Insights into progression and management

**DOI:** 10.1097/MD.0000000000042918

**Published:** 2025-06-27

**Authors:** Jie Liang, YaJun Wei, JunCai Deng

**Affiliations:** a Orthopedic Trauma Department 1 (Spinal Surgery), Chengdu Pidu District Hospital of Traditional Chinese Medicine, Chengdu, China.

**Keywords:** 3-year follow-up, intramuscular schwannoma, progression

## Abstract

**Rationale:**

Intramuscular schwannoma in the psoas major is rare and often asymptomatic in the early stages, leading to a diagnostic delay that averages 2 to 3 years in reported cases. Usually, timely treatment is required when the corresponding symptoms appear. At present, there are few studies on the preoperative follow-up of intramuscular schwannomas of the psoas major.

**Patient concerns:**

We report the case of a patient with recurrent lower back pain for 3 years, which was relieved after rest.

**Diagnoses:**

In July 2021, computed tomography revealed a tumor in the right psoas muscle, and the size of the tumor increased from 1.5 cm × 1.4 cm × 2.0 cm to 3 cm × 3.2 cm × 4 cm after 2 years and 7 months, with worsening in symptoms. The patient developed frequent swelling and pain in the right waist accompanied by pain and numbness in the right thigh root before surgery.

**Interventions:**

Based on the patient’s symptoms and the results of lumbar computed tomography and magnetic resonance imaging examinations, right lumbar muscle lateral anterior tumor resection surgery was performed under general anesthesia.

**Outcomes:**

After lateral anterior resection of the right lumbar muscle tumor, the patient’s symptoms improved significantly, and was discharged the day after surgery.

**Lessons:**

In subsequent clinical practice, we will pay more attention should be paid to patients with low back pain accompanied by nerve root symptoms. In particular, when anti-inflammatory treatment is ineffective, space-occupying lesions should be excluded. Follow-up may be reasonable, especially for elderly patients whose symptoms are tolerable and have surgical contraindications. More follow-up data are needed to help us understand the development of schwannomas in the psoas muscle. This case is of great significance for understanding the progression of intramuscular schwannomas of the psoas major.

## 1. Introduction

Schwannomas, also known as Schwann cell tumors, are derived from the Schwann sheath of the peripheral nerve. Schwannomas are clinically common, but rare cases occur in the psoas muscle, accounting for only approximately 3%.^[1]^ The clinical manifestations of intramuscular schwannoma of the psoas major lack specificity and are prone to be confused with lumbar degenerative diseases, such as lumbar disc herniation and spinal canal stenosis, leading to misdiagnosis or missed diagnosis. Ultrasonography, computed tomography (CT), and magnetic resonance imaging (MRI) are common diagnostic methods. CT manifestations: Round-like soft tissue density shadows in the psoas major muscle may be accompanied by punctate calcification or cystic changes, and destruction of the adjacent bone is occasionally observed. MRI features: low or iso-signal intensity on T1-weighted images and mixed high-signal intensity on T2-weighted images. Uneven enhancement can be seen on enhanced scans, and the “pomegranate seed” sign or ring enhancement pattern appears in some cases. The advantage of MRI lies in its ability to clearly display the anatomical relationships between the tumor and the surrounding nerves, blood vessels, and muscles, providing a basis for the selection of the surgical approach. Postoperative pathological examination is the gold standard for diagnosis, typically characterized by the coexistence of Antoni A-type (cells densely arranged) and Antoni B-type (loose mucoid matrix) structures.^[2]^

The clinical manifestations of intramuscular schwannoma of the psoas major include lower back pain, numbness of the lower limbs, intermittent claudication, and muscle weakness. These symptoms are usually associated with tumors compressing nearby nerves or the spinal cord, with very few acute symptoms such as fever, vomiting, abdominal colic, and burning urination.^[3,4]^ Surgical treatment is the primary method for treating intramuscular schwannoma of the psoas major, typically involving resection through a midline posterior or retroperitoneal approach. During surgery, it is crucial to protect the lumbar vertebrae and nerve roots to avoid damage that can lead to postoperative complications. In recent years, the application of endoscopic techniques has provided new possibilities for minimally invasive treatment, especially in complex or recurrent cases. After receiving surgical treatment, the symptoms of patients can be effectively relieved and show a good prognosis with a low recurrence rate.^[5]^

## 2. Clinical presentation

A 35-year-old male developed pain on the right side of the lower back 3 years prior, without any obvious cause. The pain worsened while walking, and the symptoms were slightly relieved when lying flat. There were no abnormalities in defecation and urination, intermittent claudication, lower limb pain, or other discomfort. After treatment with oral analgesics (the specific details are unknown), the symptoms were slightly alleviated. Lumbar muscle strain was suspected, and no further medical treatment was performed. Recently, the pain aggravated, accompanied by pain and numbness in the right thigh root, which could not be relieved after rest. The patient was admitted to the hospital on February 27, 2024. After admission, all laboratory tests (routine blood examination, liver and kidney function, coagulation, and C-reactive protein level) showed no abnormalities.

**Figure 1.**
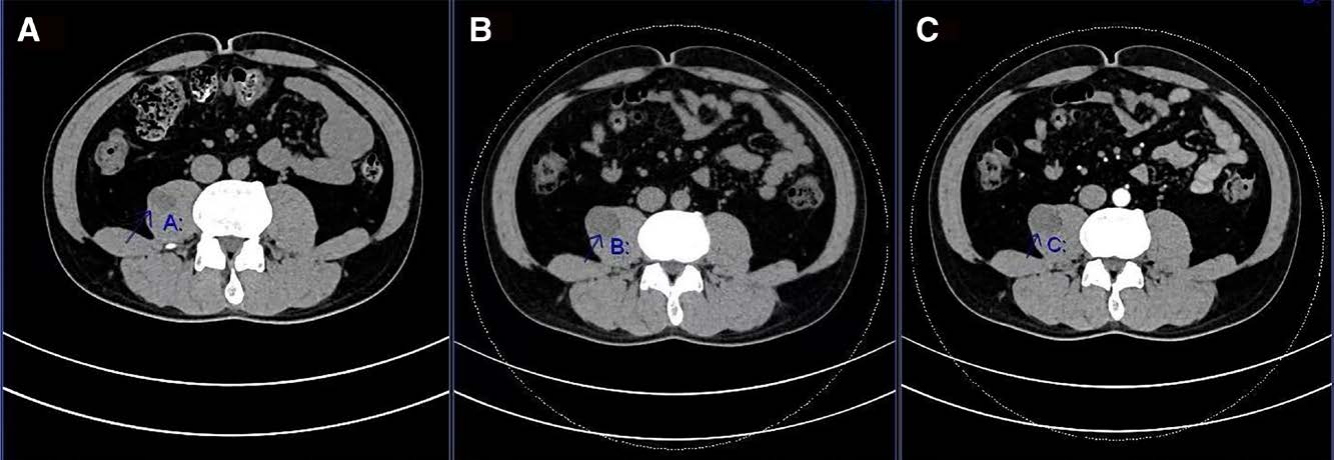
CT image. (A) in July 2021, the mass of psoas was well-defined and rounded; (B,C) in February 2024, the mass of psoas was enlarged and well-defined with no obvious blood supply. CT = computed tomography.

The patient has been followed-up in our hospital since July 2021, when the right psoas muscle mass (approximately 3/4 of the waist) was found in our outpatient department. The changes in the mass detected on CT are shown in Table 1. From the table, it can be seen that the tumor size doubled over a period of more than 2 years. The lumbar CT scan on July 16, 2021 showed a slightly hypodense shadow with the size of approximately 1.5 cm × 1.4 cm × 2.0 cm in the right psoas major muscle, with a CT value of about 33 HU and a relatively clear boundary. The enhanced CT scan on February 20, 2024, showed a mixed density shadow with a size of approximately 30 mm × 32 mm × 40 mm in the right psoas major muscle, mainly slightly hypodense, with a low-density area in the upper part and a clear boundary. The average CT value was approximately 24 HU, and there was delayed enhancement during the enhanced scan (Fig. 1).

**Table 1:** CT examination record at different time.

Time	2021-7-16	2024-2-20
The size of the tumor	1.5 cm × 1.0 cm × 2.0 cm	3 cm × 3.2 cm × 4 cm
CT values	33 HU	24 HU
Border	Clear	Clear

CT = computed tomography.

**Figure 2.**
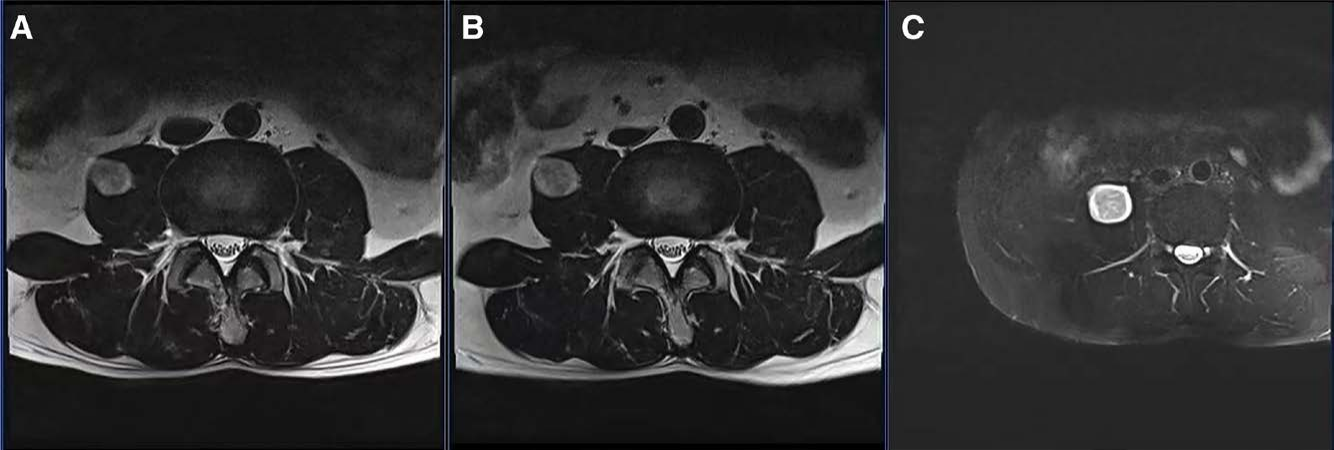
The MRI images. Panel (A) shows a slightly longer T2 signal with a diameter of about 1.7 cm in the right psoas muscle; panel (B) shows a slightly longer T2 signal with a maximum diameter of about 1.9 cm in the right psoas muscle; panel (C) shows a signal of about 30 mm × 32 mm × 40 mm T1/T2 slightly longer with clear border.

During this period, there were 3 MR examinations with values shown in Table 2. From February 2022 to January 2024, the maximum diameter of the tumor doubled in approximately 2 years (Fig. 2).

**Table 2:** MRI examination data.

Time	2021-8-4	2022-2-21	2024-1-3
Maximum	1.7 cm longer	1.9 cm longer	4 cm longer
Diameter	T2 signal	T2 signal	T2 signal
Border	Clear	Clear	Clear

MRI = magnetic resonance imaging.

### 2.1. Admission diagnosis according to imaging: right lumbar muscle tumor

**Figure 3.**
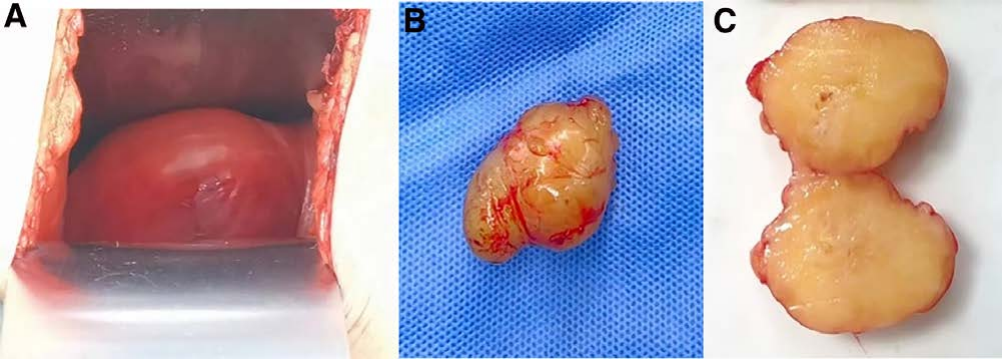
Gross (A,B) and longitudinal (C) view of the tumor. MRI = magnetic resonance imaging.

Based on the patient’s symptoms and the results of lumbar CT and MRI examinations, right lumbar muscle lateral anterior tumor resection surgery was performed under general anesthesia on March 1, 2024. The patient was placed in a left-lying position, and the surgical approach was performed along the muscle fibers to bluntly separate the iliopsoas muscle, external oblique muscle, internal oblique muscle, and transverse abdominal muscle, exposing the psoas muscle. Intraoperatively, a 3 cm × 5 cm × 3 cm well-circumscribed mass was identified within the psoas muscle, separated from the surrounding muscle fibers by blunt dissection. The tumor was resected en bloc with preservation of the overlying fascia, and intraoperative frozen section pathology confirmed the diagnosis of schwannoma (Fig. 3). The mass was elliptical in shape and gray-yellow in color, with clear boundaries, and a small pedicle connected to the surface of the capsule. After separating the small nerves, the tumor was completely removed and the wound was washed and sutured. During surgery, frozen tissue was sent for testing, which indicated schwannoma. The postoperative immunohistochemical results were the same as those for frozen tissue.

**Figure 4.**
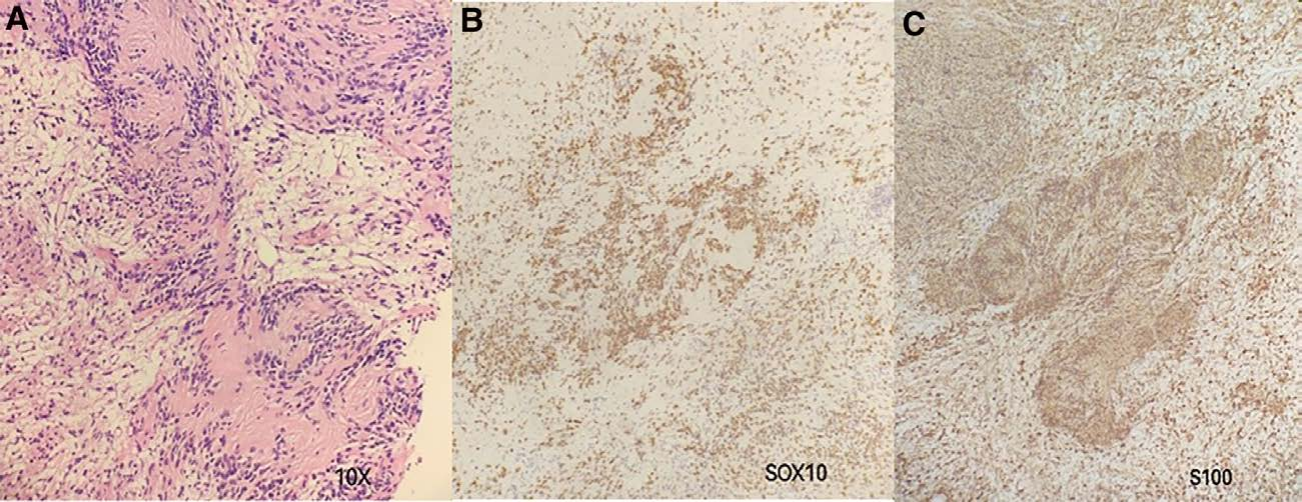
The images of the pathological examination. (A) Hematoxylin and eosin staining, ×10. Immunohistochemical staining of S0X10 (B) and S100 (C), ×10.

The hematoxylin and eosin-stained image of the pathological examination is shown in Figure 4. Histologically, benign schwannomas have capsules, and different regions present with Antoni A and Antoni B regions. Figure 4 shows that the Antoni A region is rich in cells, composed of spindle-shaped cells, with nuclei arranged in a wavy or palisade-like pattern to form Verocay bodies. The Antoni B region is composed of similar cells, but with fewer cells and a mucinous matrix. S100 protein showed strong diffuse positive staining.

**Figure 5.**
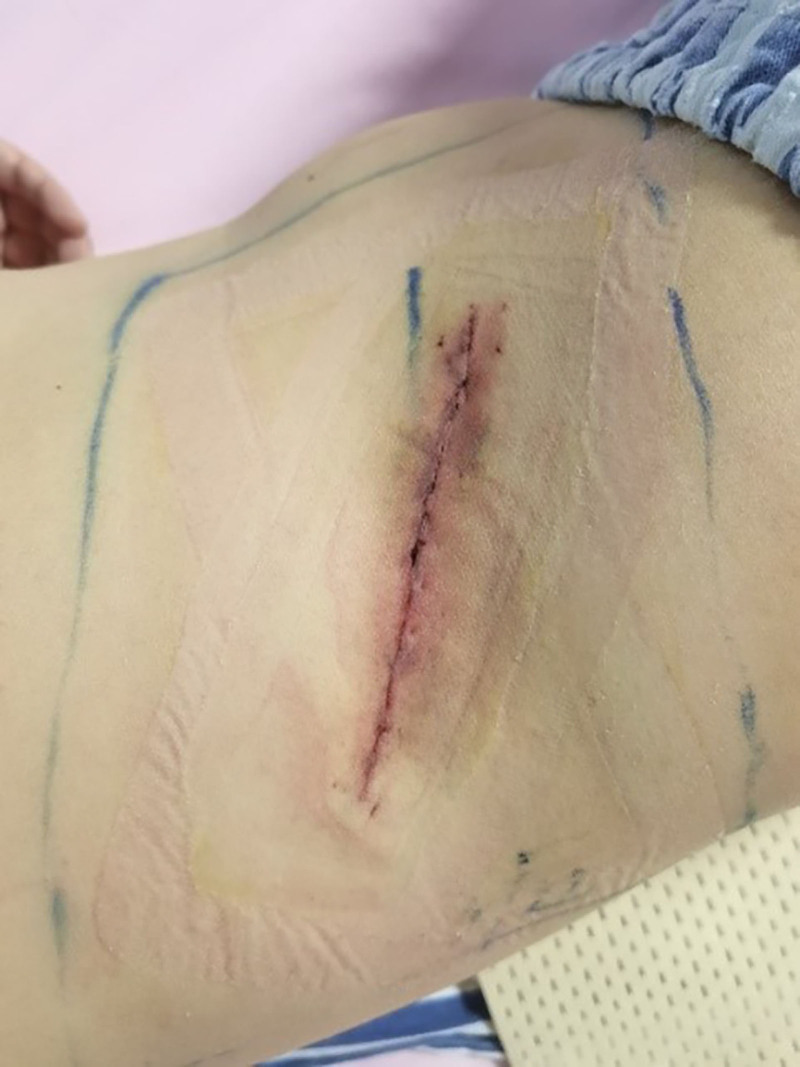
Postoperative incision.

On the second day after surgery, the patient’s symptoms of lower back pain, right thigh root pain, and numbness had disappeared. The patient was discharged on the third postoperative day, the surgical wound is shown in Figure 5. The prognosis is good. To date, the patient has not relapsed.

## 3. Discussion

Due to the insidious and nonspecific symptoms, intramuscular schwannoma in the psoas major is often overlooked. Patients may not have sought medical attention for months or years. Generally, the time of diagnosis of intramuscular schwannoma in the psoas major is closely related to symptom severity. For example, chronic pain may not be diagnosed until 15 years after its onset,^[6]^ whereas acute symptoms can lead to a diagnosis within 2 weeks.^[4]^ Currently, for the reported cases, there is usually a time gap of averages 2 to 3 years from the appearance of symptoms to diagnosis.^[6–8]^ Clinically, for patients with unexplained low back pain accompanied by nerve root symptoms, especially when anti-inflammatory treatment is ineffective, nerve sheath tumors in the psoas major muscle should be considered. When there are manifestations, such as decreased lower limb muscle strength, micturition disorders, or the appearance of abdominal masses, space-occupying lesions should be preferentially excluded. A pathological diagnosis should be initiated as early as possible.

In the reported cases, the size of the tumor also varied greatly, with diameters ranging from 3 to 20 cm. Usually, timely treatment is required when the corresponding symptoms appear. At present, there are few studies on the preoperative follow-up of intramuscular schwannomas of the psoas major. In research, the size of tumors does not indicate clinical symptoms or risk of malignancy.^[9]^ Levine et al analyzed 8 patients with schwannoma and found that the average diameter of malignant tumors was 7.9 cm, while that of benign tumors was 9.0 cm.^[10]^ However, larger tumors often exhibit high vascularization and are prone to degeneration, cystic degeneration, calcification, bleeding, and necrosis.^[11–13]^ Although tumors with uneven imaging, invasive growth, or regular bone destruction are more common in malignant tumors, they may also be present in benign schwannomas. However, malignant tumors may have smooth surfaces and noninvasive growth.^[10,14,15]^

The uniqueness of this case lies in the patient’s 3-year medical history and preoperative follow-up history of 2 years and 7 months. During this time, follow-up CT and MRI showed that the tumor had doubled in size, and the symptoms worsened accordingly. Three years ago, there was pain in the right side of the lower back, which could be relieved after rest. However, 3 months before surgery, the patient experienced frequent swelling and pain on the right side of the lower back, accompanied by pain and numbness in the right thigh root. At present, there is limited monitoring data on the development of schwannomas in the psoas muscle. In the early stages, the most common signal is asymptomatic, and retroperitoneal symptoms occur when the surrounding tissues and peripheral nerves.^[16]^ The initial symptoms of schwannoma in the psoas muscle are pain limited to the tumor site, peripheral nerve motor disorders, and sensory disorders.^[17]^ Later, when the nerve is damaged, the pain usually abates, but motor injury worse.^[18,19]^ One patient^[7]^ had a tumor measuring 42 mm × 37 mm × 60 mm with symptoms of nonspecific low back pain radiating to the left anterior calf and left ankle for several years. However, in 1 case of a giant retroperitoneal schwannoma,^[8]^ CT showed a 42 cm × 16 cm × 16 cm tumor, and only 2 years of persistent thigh pain, aggravated lower abdominal distension, and no neurologic symptoms. There was no growth during the follow-up period of 3.5 years.^[3]^

## 4. Conclusion

The symptoms of intramuscular schwannoma of the psoas major are insidious and nonspecific and are not likely to attract attention. Therefore, there is a lack of tumor data at the onset of symptoms, making it difficult to summarize the development patterns of intramuscular schwannomas of the psoas major. Even if the symptoms persist for 2 years, there was a large difference in the tumor size. The size of 1 tumor was 42 cm × 16 cm × 16 cm,^[8]^ while in another reported case, the size of the tumor was 6.4 cm × 8.5 cm × 6 cm.^^[20]^^ In subsequent clinical practice, we will pay more attention should be paid to patients with low back pain accompanied by nerve root symptoms. In particular, when anti-inflammatory treatment is ineffective, space-occupying lesions should be excluded. Follow-up may be reasonable, especially for elderly patients whose symptoms are tolerable and have surgical contraindications. Accumulating more preoperative follow-up information can help us understand the development speed of intramuscular schwannoma of the psoas major, which is of great significance for the research, diagnosis, and treatment of intramuscular schwannoma of the psoas major.^[12]^

## Author contributions

**Conceptualization:** JunCai Deng.

**Formal analysis:** Jie Liang, YaJun Wei, JunCai Deng.

**Investigation:** Jie Liang, YaJun Wei.

**Writing – original draft:** Jie Liang.

**Writing – review & editing:** JunCai Deng.
